# Spontaneous Abortion and Chikungunya Infection: Pathological Findings

**DOI:** 10.3390/v13040554

**Published:** 2021-03-25

**Authors:** Natália Salomão, Michelle Brendolin, Kíssila Rabelo, Mayumi Wakimoto, Ana Maria de Filippis, Flavia dos Santos, Maria Elizabeth Moreira, Carlos Alberto Basílio-de-Oliveira, Elyzabeth Avvad-Portari, Marciano Paes, Patrícia Brasil

**Affiliations:** 1 Interdisciplinary Medical Research Laboratory Rio de Janeiro, Oswaldo Cruz Institute, Oswaldo Cruz Foundation, Rio de Janeiro 21040-900, Brazil; natgsalomao@gmail.com; 2 Acute Febrile Diseases Laboratory, Evandro Chagas National Infectiology Institute, Oswaldo Cruz Foundation, Rio de Janeiro 21040-360, Brazil; mobrendolin@gmail.com (M.B.); mayumidw@gmail.com (M.W.); 3 Ultrastructure and Tissue Biology Laboratory Rio de Janeiro, Rio de Janeiro State University, Rio de Janeiro 20551-030, Brazil; kissilarabelo91@gmail.com; 4 Flaviviruses Laboratory, Oswaldo Cruz Institute, Oswaldo Cruz Foundation, Rio de Janeiro 21040-900, Brazil; abispo@ioc.fiocruz.br; 5 Viral Immunology Laboratory, Oswaldo Cruz Institute Rio de Janeiro, Oswaldo Cruz Foundation, Rio de Janeiro 21040-900, Brazil; flaviabarretod@gmail.com; 6 National Institute of Women, Children and Adolescents Health Fernandes Figueira, Oswaldo Cruz Foundation, Rio de Janeiro 21040-900, Brazil; bebeth@iff.fiocruz.br (M.E.M.); bethavvad@gmail.com (E.A.-P.); 7 Pathological Anatomy, Gaffrée Guinle University Hospital Rio de Janeiro, Federal University of the State of Rio de Janeiro, Rio de Janeiro 20270-004, Brazil; basiliopatologia@br.inter.net

**Keywords:** Chikungunya, pathological findings, spontaneous abortion, PCR confirmed infection

## Abstract

Intrauterine transmission of the Chikungunya virus (CHIKV) during early pregnancy has rarely been reported, although vertical transmission has been observed in newborns. Here, we report four cases of spontaneous abortion in women who became infected with CHIKV between the 11th and 17th weeks of pregnancy. Laboratorial confirmation of the infection was conducted by RT-PCR on a urine sample for one case, and the other three were by detection of IgM anti-CHIKV antibodies. Hematoxylin and eosin (H&E) staining and an electron microscopy assay allowed us to find histopathological, such as inflammatory infiltrate in the decidua and chorionic villi, as well as areas of calcification, edema and the deposition of fibrinoid material, and ultrastructural changes, such as mitochondria with fewer cristae and ruptured membranes, endoplasmic reticulum with dilated cisterns, dispersed chromatin in the nuclei and the presence of an apoptotic body in case 1. In addition, by immunohistochemistry (IHC), we found a positivity for the anti-CHIKV antibody in cells of the endometrial glands, decidual cells, syncytiotrophoblasts, cytotrophoblasts, Hofbauer cells and decidual macrophages. Electron microscopy also helped in identifying virus-like particles in the aborted material with a diameter of 40–50 nm, which was consistent with the size of CHIKV particles in the literature. Our findings in this study suggest early maternal fetal transmission, adding more evidence on the role of CHIKV in fetal death.

## 1. Introduction

The chikungunya virus (CHIKV) is an arthropod-borne arbovirus belonging to the *Alphavirus* genus and *Togaviridae* family [1]. It is an enveloped, positive-stranded RNA virus of ~11.8 kb whose genome encodes five structural proteins (capsid, E3, E2, 6K and E1) and four nonstructural proteins (nsP1–4) [2]. First described in the 1950s during an outbreak in Southern Tanzania, it has recently reemerged and, since 2014, has spread to several geographical areas that include the Caribbean and the Americas. The virus, which is transmitted by *Aedes* mosquitoes [3], principally replicates in the skin and blood, although it has been detected in muscles, joints, lymph nodes, the spleen and the brain [4]. An infection by CHIKV is characterized by a sudden onset of fever associated with joint pain that can be easily confused with infections by other arboviruses such as Dengue and Zika, which can complicate an accurate diagnosis [5]. After the early phase, 30–40% of patients may suffer recurrent joint pain that can persist for years [4].

During an epidemic on Reunion Island in 2005 and 2006, severe or complicated forms of Chikungunya were reported in adult patients, most of whom had chronic comorbidities such as hypertension and diabetes [6,7]. This period marked the first report on the vertical transmission of CHIKV [8]. The infection rate of newborns for CHIKV whose mothers displayed high viremia at the time of birth was as high as 49% [8]. In newborns, a CHIKV infection may result in a severe multiorgan involvement, along with the occurrence of fever, rash, irritability, meningoencephalitis, microcephaly and neurodevelopmental delay [9,10,11]. Additional cases of vertical transmission have subsequently been reported, which have resulted in the deaths of neonates [11,12]. In 2014, two women in Jamaica who became infected with CHIKV during the third trimester of pregnancy gave birth to newborns who died after hemorrhages that displayed hemodynamic and myocardial disorders [13]. In addition, CHIKV was associated with fetal loss between the 12th and 15th weeks of gestation [14]. IgM anti-CHIKV antibodies were detected in the mothers, and the viral genome was detected in the amniotic fluid, placenta and fetal brain by RT-PCR [14].

Vertical transmission has been described in other arboviruses, such as *Flaviviruses* including West Nile, Dengue and Zika, with viral replication in the placenta of humans and mice [15,16,17,18,19]. The alphaviruses Getah, Ross River (RRV) and Semliki Forest viruses (SFV) have shown the presence of a high titer in the placenta of mice [20,21,22]. Any changes to the placenta may lead to undesirable outcomes to the gestation and/or to the fetus/newborn, as it is a provisional organ essential for communication between the fetus and the mother [23]. The genome of CHIKV in placentas from gestating women with CHIKV infections has been reported in the literature previously [12,14,24,25]. However, little is known about the effects of virus replication in the placenta.

The objective of this report is to describe four cases of spontaneous abortions of women who became infected with CHIKV in Brazil during the first and second trimesters of pregnancy. To investigate whether the presence of CHIKV had a negative impact on the placenta and on the maintenance of the pregnancies, histopathology and ultrastructure analyses of the abortion materials were performed.

## 2. Materials and Methods

### 2.1. Ethical Procedures and Sample Collection

All procedures received prior approval by Research Ethics Committee of Oswaldo Cruz Foundation (CAAE: 81829317.3.0000.5262, approved on 10 January 2018 and CAAE: 92728218.5.0000.5248, approved on 13 October 2020). Each mother was informed of the study details and provided written consent for the publication of the data. Biological samples were collected in the Maternity ward of Hospital Estadual Adão Pereira Nunes (HEAPN) in Duque de Caxias, Rio de Janeiro, Brazil. Abortion material from a healthy patient without any infectious disease history during pregnancy was used as a negative control.

### 2.2. Clinical Case Descriptions

Case 1: A 40-year-old pregnant woman at 10 + 2 weeks of gestation of her third pregnancy was evaluated at the outpatient maternity ward of HEAPN for vaginal bleeding. She reported no symptoms other than an episode of fever three weeks earlier. On examination, she was afebrile, anicteric and not pallid. She did not present itching, rash or arthralgia. The patient was married, identified as multiracial, completed high school and was employed as a food preparation worker. She denied smoking and the use of alcohol or illicit drugs. She did not attend prenatal consultations, had no history of travel during pregnancy and was not vaccinated against yellow fever. The patient reported an episode of a Zika-like disease two years before (2016), which was not laboratory confirmed. Her blood pressure was 110 × 70 mmHg, with an axillary temperature of 36.2 °C and a heart rate 91 bpm. Moderate vaginal bleeding was observed. Upon examination, the cervix was found to be softened and the uterus enlarged. An ultrasound revealed an ectopic pregnancy of no more than five weeks’ gestation age. Respiratory and cardiovascular examinations of the mother were unremarkable. The patient was admitted with a diagnosis of incomplete abortion, and routine laboratory tests were performed. Winter curettage was performed at admission, and the collected material was sent for histopathological analysis. Laboratory tests results were RT-PCR positive for CHIKV in the urine, rapid tests for syphilis and HIV negative and a normal blood count, except for anemia (hemoglobin = 10.5 g/dL).

Case 2: A 25-year-old pregnant woman at 11 + 3 weeks of gestation was hospitalized at HEAPN with a diagnosis of spontaneous aborted pregnancy, which started 24 h before with mild vaginal bleeding and pain in the lower abdomen. She reported no symptom suggestive of CHIKV infection during pregnancy. She did not start prenatal care, did not use medication and had no comorbidities. A fetal ultrasound was performed that showed a topical gestational sac with regular contours and a large amount of amorphous echoes around, with an average diameter of 13 mm. Vitelline embryo and vesicle were not observed. A dilation of the cervix was performed with a Hegar candle and then winter curettage with moderate outflow of the ovarian remains. Laboratory tests results were IgM serological tests negative for ZIKV and DENV and positive for CHIKV; RT-PCR for CHIKV, DENV and ZIKV negative; rapid tests for syphilis and HIV negative and a normal blood count.

Case 3: A 24-year-old pregnant woman at 17 + 5 weeks of gestation was admitted at HEAPN because of premature rupture of the membranes and abortion. She reported no symptoms of CHIKV infection during pregnancy. She did not start prenatal care, did not use medication and had no comorbidities. A fetal ultrasound was performed that showed a marked oligodramnia and the absence of fetal beats. Labor was induced with misoprostol that resulted in the expulsion of the fetus weighing 238 g and leaving the placenta. It was not necessary to perform winter curettage. Laboratory tests results were IgM serological tests negative for ZIKV and DENV and positive for CHIKV; RT-PCR for CHIKV, DENV and ZIKV negative; rapid tests for syphilis and HIV negative and a normal blood count, except for anemia (hemoglobin = 10.9 g/dL).

Case 4: A 25-year-old pregnant woman at 15 + 6 weeks of gestation hospitalized with a diagnosis of abortion and endometritis after mild vaginal bleeding for the last 5 days, pain in the lower abdomen and expulsion of the fetus at home. She reported symptoms suggestive of CHIKV infection: fever, arthralgia, rash and itching. She did not start prenatal care, did not use medication and had no comorbidities. Treatment with antibiotic therapy was started: clindamycin (four days) and amikacin (three days), due to leukocytosis on admission. Winter curettage was performed, with the removal of the ovular remains in small quantities. Laboratory tests results were IgM serological tests negative for ZIKV and DENV and positive for CHIKV; RT-PCR for CHIKV, DENV and ZIKV negative; rapid tests for syphilis and HIV negative and a normal blood count, except for leukocytosis.

### 2.3. Histopathological Analysis

Abortion samples (control and Chikungunya virus infection) were collected and fixed in formalin (10%), cut into smaller fragments, dehydrated in ethanol, clarified in xylene and immersed in a paraffin resin. Tissue sections of 5 µm were obtained with a microtome. Next, tissues were deparaffinized in three baths of xylene and rehydrated with decreasing concentrations of ethanol (100% to 70%). Finally, sections were stained with hematoxylin and eosin (H&E), visualized on an Olympus DP73 light microscope (Olympus, Center Valley, PA, USA) and the digital images were obtained using Olympus cell Sens Standard.

### 2.4. Quantitative Analysis

Histopathological changes, such as (i) fibrinoid material, (ii) inflammatory infiltrate and (iii) edema, were quantified in infected placenta tissue compared to the control. For this, fifteen digital images (magnification 400×) were captured, and an arbitrary scale was used to characterize the damage: 0—absent, 1—light and focal, 2—light, 3—moderate and 4—diffuse.

### 2.5. Immunohistochemistry Techniques

The paraffin-embedded tissues (4-µm-thick units) were deparaffinized in xylene and rehydrated with alcohol. Antigen retrieval was performed by heating the tissue in the presence of a citrate buffer. The tissues were blocked for endogenous peroxidase with 3% hydrogen peroxidase in methanol and rinsed in Tris–HCl (pH 7.4). Next, the tissues were incubated in Protein Blocker solution (Spring Bioscience, Pleasanton, CA, USA) for 10 min at room temperature to reduce nonspecific binding. Tissue samples were incubated overnight at 4 °C with a monoclonal anti-CHIKV antibody (1:10) (Novus Biologicals—3E7b, Centennial, CO, USA) and polyclonal anti-CHIKV mouse hyperimmune ascites fluids. On the following day, the tissue was incubated with a secondary complement (REVEAL complement—Spring Bioscience, Pleasanton, CA, USA) for 10 minutes and with a rabbit anti-mouse IgG-HRP conjugate (REVEAL polyvalent HRP—Spring Bioscience) for 15 min at room temperature. Reactions were revealed with diaminobenzidine (Spring Bioscience) as a chromogen, and the sections were counterstained with Harris hematoxylin (Wcor Corantes, Guarulhos, SP, Brazil).

### 2.6. Electron Microscopy Assay

Tissue samples were fixed with 2.5% glutaraldehyde in a sodium cacodylate buffer (0.1 M, pH 7.2); postfixed with 1% buffered osmium tetroxide (Electron Microscopy Sciences, Hatfield, PA, USA); dehydrated in an acetone series (30%, 50%, 70%, 90% and 100%—Sigma, St. Louis, MO, USA) and embedded in EPON (electron microscopy) that was polymerized at 60 °C for 3 days. Ultrathin sections (60–90 nm) were obtained using a diamond knife (Diatome, Biel, Switzerland) adapted to a Reichert-Jung Ultracut E microtome (Markham, ON, Canada). Sections were contrasted with uranyl acetate and lead citrate (electron microscopy) before visualization on a JEOL 1001 transmission electron microscope (Jeol, Tokyo, Japan).

## 3. Results

### 3.1. CHIKV Infection Led to an Inflammatory Environment

**Figure 1:**
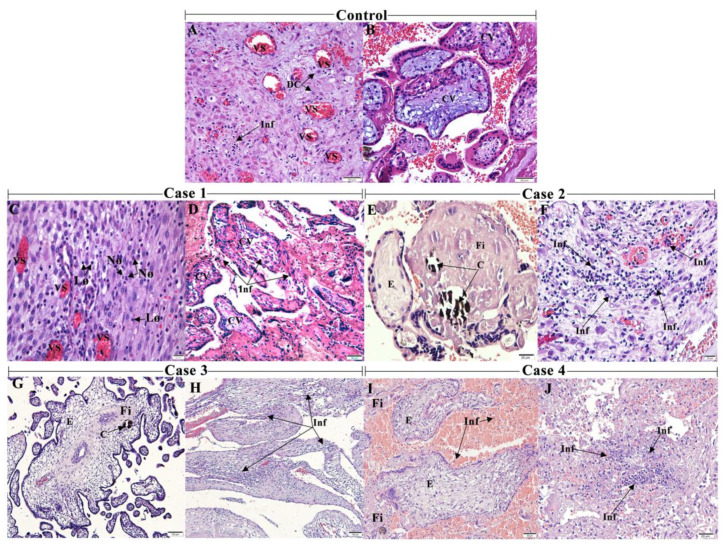
Histopathological analysis of the aborted materials detected an inflammatory environment: (**A**) abortion material from a healthy patient without any infectious disease history during pregnancy (control) showing decidual cells (DC) and (**B**) decidual vessels (DV) with the regular aspects. (**C**) Case 1 exhibiting decidua with mononuclear and polymorphonuclear—lymphocytes (Ly) and neutrophils (Nø) (**D**) and decidua with inflammatory infiltrate (Inf). (**E**) Case 2 with villous edema (E), the deposition of fibrinoid material (Fi) and calcification (C) (**F**) and decidua with intense inflammatory infiltrate. (**G**) Case 3 with dysmorphic villi with edema, focal areas of the deposition of fibrinoid material and calcification (**H**) and deciduitis. (**I**) Case 4 with intervillous space with inflammatory cells, villous edema and areas of the deposition of fibrinoid (**J**) and deciduitis. 10 µm = 1000×, 20 µm = 400×, 50 µm= 200×, 100 µm = 100×.

The spontaneously aborted biological samples consisted of the decidua, the functional endometrial layer of a pregnant woman, and the chorionic villi, the fetal portion. In the control placenta, the decidual cells and vessels of the parenchyma were more organized (Figure 1A,B). In the decidua of case 1, there was an extensive area of inflammatory infiltrate, mainly composed of lymphocytes and neutrophils, characterized by its morphology in the infected tissue. The former had a spherical nucleus with condensed chromatin and sparse basophilic cytoplasm, whereas the latter were spherical with a segmented nucleus (Figure 1C). An intense inflammatory infiltrate was also observed in the decidua from case 2 (Figure 1F), case 3 (Figure 1H) and case 4 (Figure 1J). The chorionic villi exhibited alterations such as inflammatory infiltrate in case 1 (Figure 1D) and areas of calcification, edema and deposition of the fibrinoid material in case 2 (Figure 1E) and in case 3, with dysmorphic villi (Figure 1G). The chorionic villi of case 4 also exhibited areas of fibrin deposit and edema, except for calcifications and inflammatory infiltrate (Figure 1I). In addition, the intervillous space presented lymphocytes infiltrate (Figure 1I).

**Figure 2:**
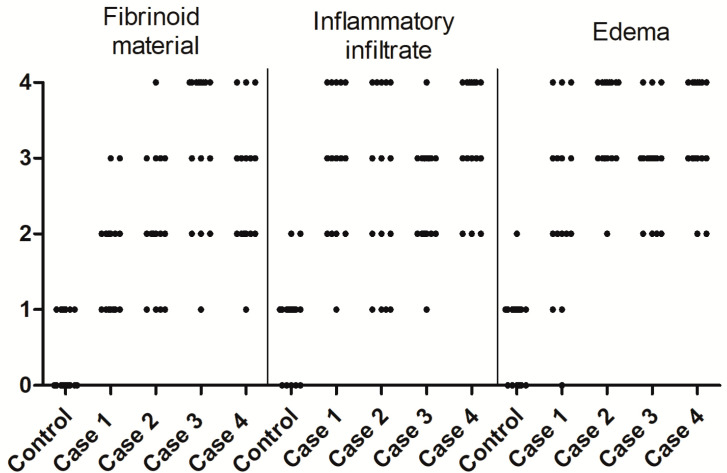
Quantification of the histopathological alterations. Fibrinoid material, inflammatory infiltrate and edema were quantified in the placental tissue of the control and cases 1, 2, 3 and 4 using an arbitrary scale with the following degrees: 0—absent, 1—light and focal, 2—light, 3—moderate and 4—diffuse.

A subjective quantification was performed using an arbitrary scale. For example, the fibrinoid material was diffuse mainly in case 3, whereas, in cases 1, 2 and 4, it was light. The deposition of the fibrinoid material, inflammatory infiltrate and edema were absent or light and focal in the control tissue placenta (Figure 2).

### 3.2. CHIKV Antigen Detection in Abortion Material

**Figure 3:**
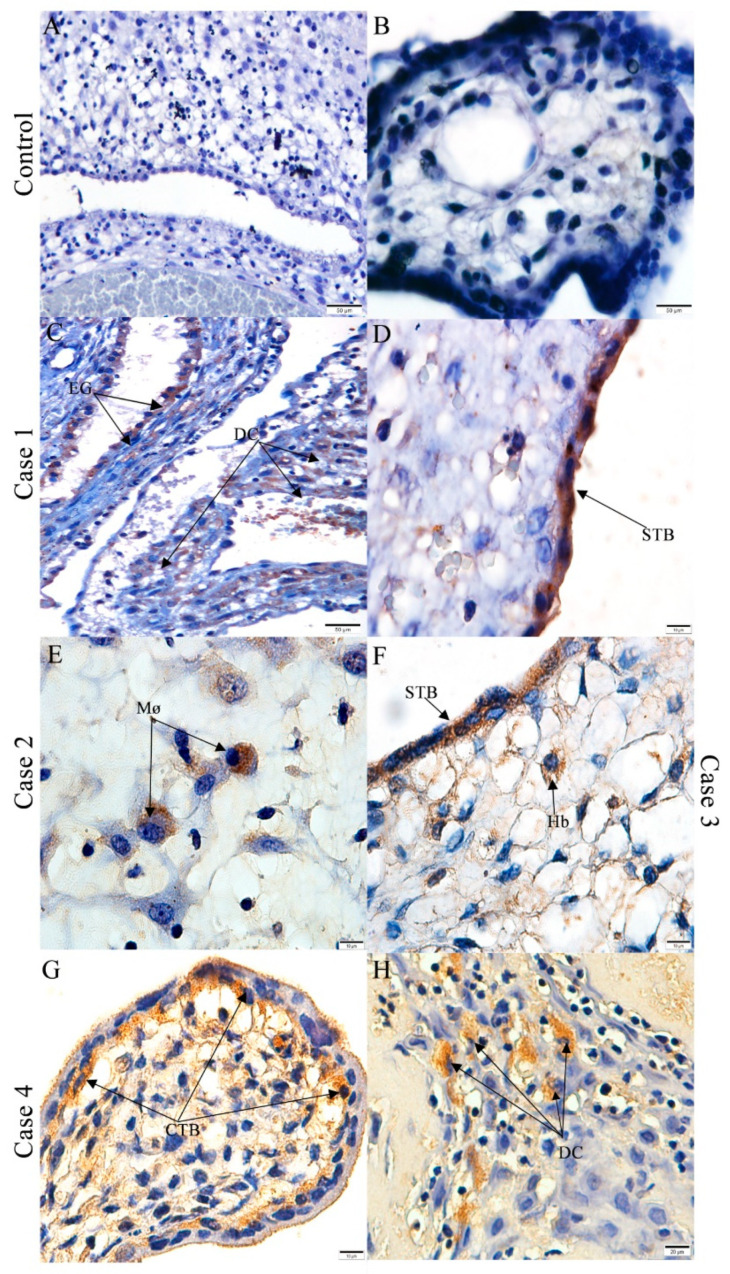
Detection of the chikungunya virus (CHIKV) antigens in aborted materials: Control aborted material without the detection of CHIKV in the (**A**) decidua and (**B**) chorionic villi. CHIKV detection in (**C**) cells of the endometrial glands (EG), decidual cells (DC) and (**D**) syncytiotrophoblasts (STB) of case 1; (**E**) decidual macrophages (Mø) of case 2; (**F**) in the syncytiotrophoblasts and Hofbauer cells (Hb) of case 3 and in (**G**) the cytotrophoblasts and (**H**) decidual cells of case 4. 10 µm = 1000×, 20 µm = 400×, 50 µm= 200×, 100 µm = 100×.

Using immunohistochemistry techniques, CHIKV antigen was detected in the in epithelial cells from the endometrial glands and in the decidual cells of case 1 (Figure 3C). Furthermore, the CHIKV antigen also was detected in some syncytiotrophoblasts of the chorionic villi (Figure 3D). In case 2, a positive immunodetection of the CHIKV antigen was seen in the decidual macrophages (Figure 3E). In case 3, the Hofbauer cells and syncytiotrophoblasts displayed reactivity for the CHIKV antigen (Figure 3F). Positive signals were observed in the cytotrophoblasts (Figure 3G) and decidual cells (Figure 3H) of case 4. As expected, no viral antigen detection was observed in the control (Figure 3A,B).

### 3.3. Ultrastructure of the Abortion Material

**Figure 4:**
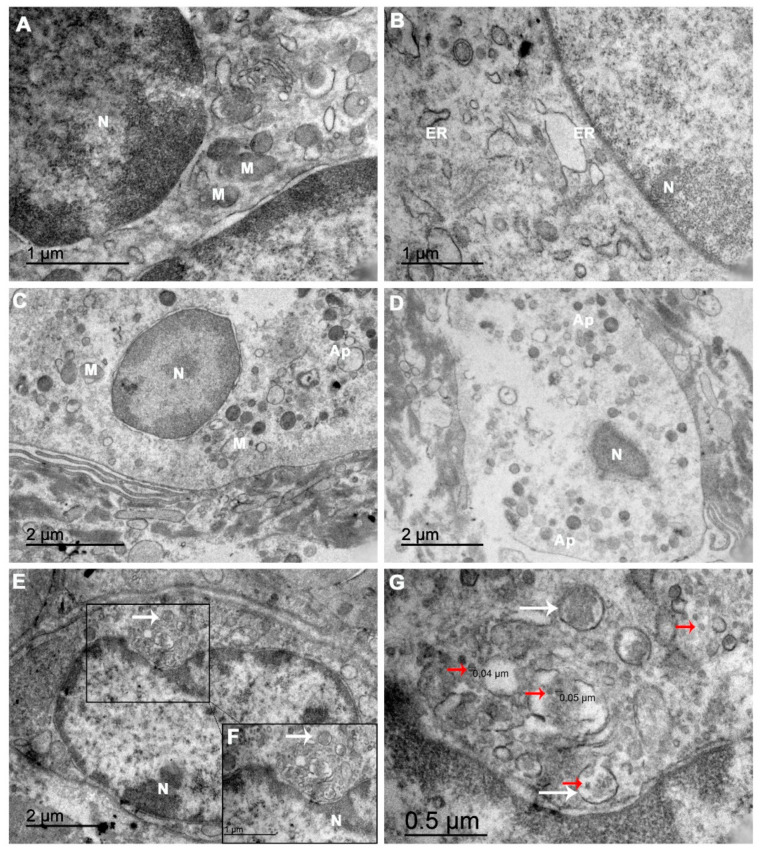
An electron microscopy analysis of the ultrathin abortion material sections showed damaged organelles and virus-like particles: (**A**) Electron micrographs of CHIKV-infected cells showing dispersed chromatin in the nuclei (N), mitochondria (M) with fewer cristae and (**B**) endoplasmic reticulum (ER) exhibiting dilated cisterns. (**C**) A decidual cell presenting a rarefied cytoplasm with an absence of organelles and starting to produce apoptotic bodies (Ap). (**D**) A decidual cell in apoptosis. (**E**,**F**) A cell with vesicles surrounding a cluster of virions in the cytoplasm (white arrows). (**G**) In the same field, at a higher magnitude, these CHIKV virus-like particles (red arrows) are located near a ruptured ER. The scale bar indicates that the particles are approximately 40–50 nm in size, consistent with the CHIKV.

The abortion material of case 1 was processed for examination by transmission electron microscopy. We noted considerable changes in the ultrastructure of the decidual cells. Multiple organelles exhibited alterations, such as the mitochondria, with fewer cristae and ruptured membranes (Figure 4A,C), and the endoplasmic reticulum (ER) with dilated cisterns (Figure 4B), as well as dispersed chromatin in the nuclei (Figure 4A–C). Some decidual cells also had rarefied cytoplasm with an absence of organelles and produced apoptotic bodies (Figure 4C), while other cells were in a more advanced stage of apoptosis (Figure 4D). Furthermore, ultrathin sections of the tissue permitted the identification of clusters with dense virus-like particles located in damaged cytoplasmic vesicles of the decidual cells (Figure 4E–G). These virus-like particles were located near disrupted areas of the ER and were approximately 40–70 nm in diameter (Figure 4G), which is consistent with the physical dimensions of CHIKV.

## 4. Discussion

Since the CHIKV epidemic on Reunion Island in 2005 and 2006, the vertical transmission of the virus has been explored. Some studies have reported newborns with symptoms similar to adults. Moreover, some authors have reported cases of fetal loss [14]. A spontaneous abortion is defined as the death of the fetus or passage of products of conception (fetus and placenta) before 20 weeks of gestation [26], which can be further classified as early (before 12 weeks of gestation) and late (between 12 and 20 weeks of gestation) [26]. Each of the cases included in this study met this definition, with one early and three late. In case 1, it was possible to detect the virus genome in the urine by RT-PCR. For the other three cases, a diagnosis was made by the presence of anti-CHIKV IgM antibodies by serology.

The age of the expecting mother is an important factor to the occurrence of spontaneous abortions [27], which could have contributed to case 1, being a 40-year-old women. However, it is worth noting that the mother did not smoke, drink alcohol, consume illicit drugs and did not present changes in blood pressure or cardiovascular function. In all cases, extensive areas of inflammatory infiltrate were observed in both the villitis and deciduitis, which may have contributed to a lack of balance in the immune response against the pathogen, leading to spontaneous abortion [28]. Moderate and diffuse inflammatory infiltrate was noted in all four cases, mainly characterized by macrophages, lymphocytes and neutrophils in the decidua. In case 4, an area of necrosis was observable. The presence of inflammatory cells is normal in aborted materials, but what we observed in the cases appeared greater in comparison to the control.

In addition, irregularities were seen in the chorionic villi that were more apparent in cases 2, 3 and 4. These included (i) edema recognizable by open spaces within the cytoplasm of intervillous cells and in the interstitium of the villi, which can be a possible cause of antenatal hypoxia [29], (ii) a notable deposition of fibrinoid material in the intervillous space that is not as common in early placentas, although it increases as a pregnancy progresses [30], and (iii) calcification from the deposition of calcium-phosphate minerals in the placenta tissue [31], which, in an early preterm placenta, is associated with a higher incidence of an adverse pregnancy outcome [32]. The characteristics of edema, the deposition of fibrinoid material and calcification have been described in the placental tissue of spontaneous abortion due to Zika virus infection [33].

Particularly, in case 2, all the chorionic villi were avascular (grade III vascularization), which is known as hydropic degeneration [34], in addition to the calcification and deposition of the fibrinoid material, which may be related to fetal thrombotic vasculopathy [35,36]. This could have impaired the exchanges between the mother and fetus, as it is the area with their greatest contact [23]. The deposition of the fibrinoid material in the intervillous space can lead to atrophy of the engulfed villi [37]. In case 3, dysmorphic villi were characterized by an irregular contour, which may have been a cause of the developmental abnormalities of the fetal stromal–vascular compartment of the placenta [38]. These findings all suggest that the CHIKV infection could have contributed to the occurrence of these spontaneous abortions.

In case 1, CHIKV antigens were detected in the epithelial cells of the endometrial glands, which are an important source of nutrients, carbohydrates and lipid-rich secretions that promote placentation. This process could have been hampered due to the CHIKV infection, which may lead to a spontaneous abortion [39]. In the placenta of cases 1, 2 and 4, it was possible to detect CHIKV antigens in both the maternal portions (cells and decidual macrophages). The CHIKV antigen was observed in the fetal portions of cases 1, 3 and 4 in the cytotrophoblast, syncytiotrophoblast and Hofbauer cells, which suggests a role for these cells in virus replication, which was also seen in Zika virus infections [18,40]. Although the syncytiotrophoblast has not been shown to be susceptible to CHIKV in vitro [41], the CHIKV antigen was detected in utero. Syncytiotrophoblast cells produce human chorionic gonadotropin (hCG), a hormone important for stimulating the corpus luteum to continuously produce progesterone, which is needed to maintain a pregnancy. Once infected, the production can altered, leading to an abortion [42].

In case 1, ultrastructural abnormalities of the cells that made up the aborted material were observed as mitochondrial changes and a dilation of the endoplasmic reticulum cisternae, which were similar to those observed in ZIKV placental infection [18]. These types of alterations are suggestive of an imbalance in protein synthesis, as well as in the energetic machinery of the cell. Furthermore, there was a correlation between the mitochondrial alterations and the cells found in the process of apoptosis. Our results suggest that CHIKV induced apoptosis in the maternal portion of the aborted material, which may have been an important step in the process that culminated in a spontaneous abortion. Moreover, the identification of virus-like particles (VLPs) with diameters between 40 and 50 nm in the samples of the ultrathin aborted material was consistent with the diameter of CHIKV virion reported in the literature that ranged from 40 to 70 nm [43,44,45,46,47].

The major limitation of this study was the absence of a molecular detection of the CHIKV genome in the examined tissues. It is possible that the collection and storage were not sufficiently rigorous to preserve the viral RNA and that it suffered degradation. Through the immunohistochemistry technique, we could affirm that CHIKV reached the placental sites. However, it was not indicative of viral replication, since we did not use any nonstructural protein antigen. Conversely, the wrapped VLPs in the ruptured ER suggested that there was viral replication in the decidual cells. Together, our results provide further evidence of the vertical transmission of CHIKV early in pregnancy in asymptomatic and symptomatic cases (50%). The present findings are also indicative of a direct link between CHIKV infection and fetal death, as there was no evidence of other maternal morbidities. Nevertheless, further studies are needed to elucidate the mechanisms by which CHIKV disrupts a pregnancy to avoid unfavorable obstetric and neonatal outcomes in the future.

## Data Availability

All data generated or analyzed during this study are included in this published article.
